# Advances in paediatrics in 2016: current practices and challenges in allergy, autoimmune diseases, cardiology, endocrinology, gastroenterology, infectious diseases, neonatology, nephrology, neurology, nutrition, pulmonology

**DOI:** 10.1186/s13052-017-0401-9

**Published:** 2017-09-16

**Authors:** Carlo Caffarelli, Francesca Santamaria, Dora Di Mauro, Carla Mastrorilli, Silvia Montella, Sergio Bernasconi

**Affiliations:** 1 Clinica Pediatrica, Department of Medicine and Surgery, Azienda Ospedaliera-Universitaria, University of Parma, Via Gramsci, 14 Parma, Italy; 2 Department of Translational Medical Sciences, Federico II University, Naples, Italy; 3 Pediatrics Honorary Member University Faculty, G D’Annunzio University of Chieti-Pescara, Chieti, Italy

**Keywords:** Allergy, Autoimmune diseases, Cardiology, Childhood, Endocrinology, Gastroenterology, Infectious diseases, Neonatology, Nephrology, Neurology, Nutrition, Pulmonology

## Abstract

This review reports main progresses in various pediatric issues published in Italian Journal of Pediatrics and in international journals in 2016. New insights in clinical features or complications of several disorders may be useful for our better understanding. They comprise severe asthma, changing features of lupus erythematosus from birth to adolescence, celiac disease, functional gastrointestinal disorders, Moebius syndrome, recurrent pneumonia. Risk factors for congenital heart defects, Kawasaki disease have been widely investigated. New diagnostic tools are available for ascertaining brucellosis, celiac disease and viral infections. The usefulness of aCGH as first-tier test is confirmed in patients with neurodevelopmental disorders. Novel information have been provided on the safety of milk for infants. Recent advances in the treatment of common disorders, including neonatal respiratory distress syndrome, hypo-glycemia in newborns, atopic dermatitis, constipation, cyclic vomiting syndrome, nephrotic syndrome, diabetes mellitus, regurgitation, short stature, secretions in children with cerebral palsy have been reported. Antipyretics treatment has been updated by national guidelines and studies have excluded side effects (e.g. asthma risk during acetaminophen therapy). Vaccinations are a painful event and several options are reported to prevent this pain. Adverse effects due to metabolic abnormalities are reported for second generation antipsychotic drugs.

## Background

Several papers have been recently published that can potentially alter traditional approach of many diseases in childhood. In this review, we focus on some of the new key findings in allergy, autoimmune diseases, cardiology, endocrinology, gastroenterology, infectious diseases, neonatology, nephrology, neurology, nutrition, pulmonology in childhood. We mainly have summarized articles issued in Italian Journal of Pediatrics in 2016. A choice of valuable information that appeared in international journals have also been covered and placed in context.

## Review

### Allergy. 1-Management of atopic dermatitis; 2-severe asthma; 3-role of vitamin D in asthma

Asthma [1] and atopic dermatitis (AD) are common atopic conditions that can be successfully controlled by providing patients instructions on the diseases and by explaining how to use reliever and preventive treatments. A small proportion, probably less than 5%, of asthmatic children suffers from severe asthma. This subset of asthmatics is difficult to treat and children have more frequent hospitalizations for asthma, emergency department visits for asthma, exercise-induced symptoms, and receive more commonly systemic steroids for exacerbations than non-severe asthmatics [2]. Quality of life is reduced. New insights in characteristics of children with severe asthma which present different phenotypes and endotypes [3] may provide a basis for improving treatment. Airflow obstruction is a criterion for defining asthma severity [4]. Montella et al. [2] confirmed that severe asthmatics have impaired lung function. However, they found forced expiratory volume in 1 second was normal in approximately half the cases. Accordingly, it has been shown that lung function did often not correlate with asthmatic symptoms [5] or oxidative stress [6]. Among risk factors, Montella et al. [2] reported that severe asthma was also associated with food sensitization but not with environmental exposure, comorbidities, medication adherence, exhaled nitric oxide levels, blood eosinophils and serum IgE levels. Asthmatics with vitamin D deficiency have been found to be not at risk for severe asthma [7]. However, the presence of asthma was strongly associated with low vitamin D levels in serum [7, 8]. Treatment of asthma with vitamin D in schoolchildren was the aim of a randomized, double-blind, placebo-controlled trial [9]. It has been found that 800 IU/day of vitamin D3 supplementation for 2 months significantly improved childhood asthma control test score and reduced number of asthmatic children with a peak expiratory flow rate < 80% predicted. In contrast, an initial bolus of vitamin D (100,000 IU once), followed by 4000 IU/die for 7 months did not show to be of benefit in asthmatics [10]. Tachimoto et al. [9] suggested that vitamin D supplementation at low dose may ameliorate the control of asthma in childhood.

Regarding AD, this year has been issued the Consensus on clinical management by the Italian Society of Pediatric Allergology and Immunology and the Italian Society of Pediatric Dermatology [11]. Authors recommend skin emollients and bathing in lukewarm water with non-soap cleansers and synthetic detergents with no preservatives and perfumes to maintain the epidermal barrier function. They pointed out that topical corticosteroids are the first-line therapy. In areas where relapsing is frequent, control of disease improves by applying topical corticosteroids twice a week. The document emphasizes that topical calcineurin inhibitors are a second-line option for acute flares when topical corticosteroids are not effective, contraindicated or induce side-effects. In chronic AD, they can be used as continuous or intermittent long-term treatment for 12 months, then a reassessment is warranted. Topical calcineurin inhibitors should not be used in young children less than 2 years old, congenital or acquired immunodeficiency, skin infections, eroded and/or exuding lesions, sun exposure. Wet wrap therapy or phototherapy should be considered when lesions are resistant to topical anti-inflammatory treatment. Several systemic treatments are used as a third-line option. Systemic corticosteroids can be used for short-term treatment of severe AD, while the long-term use is limited by side-effects and exacerbation after cessation of treatment. Systemic immunosuppressants including ciclosporin, azathioprine, methotrexate are used off-label apart from ciclosporin, in some European countries [12]. Optimal dose and length of treatment of azathioprine and methotrexate are unclear. Among other therapies, first-generation antihistamines may be used when nocturnal sedation is a desirable effect. Allergen immunotherapy to mites may be of benefit in a subgroup of patients [13].

### Autoimmune diseases. Lupus erythematosus during childhood

Many studies have documented the changing features of lupus erythematosus during childhood. Neonatal lupus erythematosus (NLE) is an uncommon autoimmune disease characterized by cutaneous lupus lesions and/or congenital heart block. It is due to maternal antinuclear antibodies and extractable nuclear antigen antibodies. These maternal autoantibodies against RNA protein complex, Ro/SSA or SSB/La cross the placenta and potentially lead to fetal tissue damage and the clinical manifestations NLE. The cutaneous lesions may be present at birth, but more often develop within the first few weeks of life. Savino et al. [14] presented four cases of cutaneous NLE, two females and two males, who had no signs or symptoms at birth and they developed cutaneous lesions from 2 days to 3 months after delivery. Mothers were positive for Ro/SSA and La/SSB in all the cases and one of them also presented a positivity of anti-CCP and Rheumatoid factor, with a reduction in C4. These cases confirmed that NLE is a condition usually benign and self-limited. Its evolution is the spontaneous regression of the lesions within six months without progression to systemic lupus erythematosus (SLE). Furthermore, it is common to see that autoimmunity in the mother is not detected until the birth of a child with NLE. Childhood-onset SLE is a multisystem, autoimmune disease, beginning before the age of 18 years that may affect any organ. Children are known to have a more severe disease course. Approximately one third of children will present with life-threatening, “non-classical” clinical manifestations that are not included in the revised American College of Rheumatology classification criteria. Fatemi et al. [15] retrospectively studied 188 children, 33 were diagnosed with SLE when they were <12 years, 149 (81.9%) with SLE at 12–18 years of age, 6 patients were lost to follow-up. Both groups had similar clinical manifestations and laboratory findings. During follow-up, 20 children died. The SLE group aged 12–18 years presented higher rates of deaths (13/20, 65%), followed by those aged >18 years (6/20, 30%) and those younger than 12 years (1/20, 5%). 50% of deaths occurred during the first year after SLE onset and only 25% after the first 5 years. The most common cause of death was lupus nephritis and infections (10 patients, 50% and 7 patients, 35% of all deaths) with a significant pattern of death: 53% of deaths during the first 5 years were due to infections, but all deaths after the first 5 years were due to lupus nephritis (*P* = 0.04). The overall 5, 10, 15 and 20 years survival rates after SLE onset were 91%, 87%, 85% and 79%, respectively. Age of SLE onset had no association with survival but having hematuria or pleurisy at the time of SLE onset had a negative effect on survival in multivariate analysis. The survival rate of SLE children seems to be comparable throughout the world.

Low vitamin D serum levels are common in patients with SLE and seem to correlate with higher disease activity. Lima et al. [16] conducted a randomized, double-blind, placebo-controlled, 24-week trial to investigate the effects of vitamin D supplementation on disease activity and fatigue in juvenile-onset SLE. 40 female patients were randomized into 2 parallel groups to receive oral cholecalciferol of 50,000 IU/week (SLE-VitD group) or identical placebo tablets for 6 months (SLE-PL group). Fatigue and disease activity were assessed using the Kids Fatigue Severity Scale and the Systemic Lupus Erythematosus Disease Activity Index (SLEDAI) and the European Consensus Lupus Activity Measurement (ECLAM) respectively. Demographic characteristics, clinical and laboratory parameters, medication use, including mean daily dose of glucocorticoids and the use of immunosuppressive drugs and adherence to vitamin D supplementation were assessed at baseline, after 3 months and at the end of supplementation. At baseline, the mean 25(OH)D serum levels were similar in the 2 groups and 95% of all juvenile-onset SLE patients had a vitamin D insufficiency (25(OH)D < 30 ng/ml). After 12 months, values above 30 ng/ml were found in 70% of patients in SLE-VitD group while in 0% in SLE-PL group. A significant difference in disease activity scores (SLEDAI and ECLAM) was found at 6 months compared to baseline in the juvenile-onset SLE-vitamin D group compared to the SLE-PL group (improvement in SLEDAI scores *p* = 0.011 and in ECLAM: *p* = 0.006). At the end of the study, SLE-VitD group had a better global score for fatigue than the placebo group (*P* = 0.012) and this improvement was more significant in social life fatigue score (*P* = 0.008). This study showed that routine screening for vitamin D deficiency and its prompt treatment in patients with SLE is recommended and may decrease disease activity scores and fatigue.

### Cardiology. 1-Risk factors for congenital heart defects; 2-origin of Kawasaki disease; 3-hypertension

Recent studies about congenital and acquired heart defects, mainly Kawasaki disease (KD), in childhood have evoked a great interest. Maternal alcohol consumption is associated with the fetal-alcohol syndrome that can provoke different degrees of embryopathy, ranging from minor to severe symptoms. Furthermore, it can be related with neurobehavioral disorders listed in the Diagnostic and Statistical Manual of Mental Disorders, Fifth Edition (DSM-5) as a condition for further study [17]. Children and adolescents prenatally exposed to alcohol may have brain damage with functional impairment of neurocognition, self-regulation, and adaptive functioning [18]. Up to one-third of individuals affected by fetal-alcohol syndrome have congenital heart defects (CHDs), e.g. conotruncal defects, left ventricular outflow track defects, septal defects. Several risk factors of CHDs have been recognized such as chromosomal abnormalities, maternal illnesses, prenatal exposures to teratogens. Several studies have assessed the relation between CHDs and prenatal alcohol consumption, reporting different results. Therefore, it is worthwhile that a meta-analysis by Wen et al. [19] found no association between maternal alcohol consumption during pregnancy and the risk of CHDs. These findings confirmed conclusions of a previous systematic review [20]. The major cause of acquired heart disease in developed countries is represented by coronary artery abnormalities, that is the main complication of KD. The etiology of KD remains unknown though infection is considered one of the predisposing factors. No infectious agent has been identified as unique cause of KD. Independent risk factors for coronary artery abnormalities include male gender, serum albumin, erythrocyte sedimentation rate (ESR), *Mycoplasma pneumoniae* (MP) infection, intravenous immunoglobulin (IVIG) started after the 10th day of illness and IVIG non-responders [21]. A well-designed prospective case-control study investigated the etiologic role of MP in KD [22]. MP infections were found in 62/450 KD patients and occurred in older populations and with a higher rate of respiratory tract involvement. No statistical difference in the incidence of coronary artery lesion was observed between the MP+ and MP- KD patients. Lee et al. [23] also showed no difference in both left and right coronary artery abnormalities of MP+ and MP- KD patients by analyzing 12 KD patients and 42 controls. Overall, these findings suggest that other factors different from MP should be investigated for the onset of coronary lesions in KD. Primary hypertension represents a growing problem in children and adolescence. Recent novelties in the field of prevention, diagnosis and treatment of hypertension in children were considered by Strambi et al. [24]. Authors emphasized that the definition of normal blood pressure values in neonates are lacking [25]. However, it is advisable to use results of the study by Dionne et al. [26] that provided blood pressure values corresponding to different percentiles at 15 days of age according to gestational age. In pediatric age, diagnosis of hypertension is based on repeated office measurements exceeding reference values [24]. Ambulatory blood pressure monitoring can be useful in children older than 10 years of age to clarify critical issues, such as hypertension during hypersensitivity reaction [27]), white coat hypertension, masked hypertension, treatment efficacy, nocturnal drop [24]. Strambi et al. [24] considered also another key point that is the positive effect of physical exercise on prevention of primary hypertension by reducing overweight, inferior quality of sleep and by maintaining sodium homeostasis [28]. However, they [24] reported a list of isometric and intensive sports that are not recommended in case of stage 2 hypertension not pharmacologically controlled. It must be also considered that diagnosis of hypertension should not be neglected in athletic adolescents [29].

### Endocrinology. 1- Staminal’s Research in prevention/treatment of type 1 diabetes, 2-use of Omnitrope

A series of innovative papers have addressed key topics such as management of diabetes and use of growth hormone (GH) for short stature. Replacement of pancreatic beta-cells represents an ideal treatment that could overcome the difficulties to manage multi-injective insulin and progressive worsen of diabetes [30]. Due to shortage of organs and the inability of islet to be expanded ex vivo, this therapy can be offered to a very limited number of patients. The advent of stem cells as renewable source of cell-substitutes to replenish the beta-cell population, has blurred the hype on islet transplantation. Unfortunately, a cell source providing a robust response after differentiation as embryonic stem cells (ESC), is not always considered safe because of transplant-related collateral effects such as immune rejection or, worse, tumorigenesis. For these reasons, scientists are focusing on various sources of stem cells that possess less stemness potential compared to ESC but are safer and free of ethical concerns, namely adult stem cells. The pancreas itself is a promising source for stem cells: duct cells, acinar cells and stem cell from islets of Langerhans share the same embryological origin of beta-cells; therefore, they are good candidates for the de-differentiation and re-programming towards an insulin producing phenotype. The obstacles to the extensive use of this source are represented by the invasive collection procedures, the scarcity of the stem cells, the difficulty in isolating and expanding the culture in vitro [31]. The derivation of insulin producing cells from hepatocytes (trans-differentiation or lateral re-programming), which share with beta-cells the same endodermic origin, showed promising outcomes. However, even though these tissues represent an abundant pool of reprogrammable cells, the use of hepatocytes ex vivo is hampered by the shortage of donors, poor in vitro expansion capacities, and rapid cell de-differentiation. Mesenchymal stem cells (MSC) from various tissues, pancreas included, are a widely accessible source of insulin producing cells. The expectations on MSC studies were nurtured by the abundance of cells, the ease in isolation and in vitro expansion, and the promising outcomes of diverse studies showing the differentiation potential towards non-mesodermic lineages [32]. Stem cells isolated from the amnion layer of placenta meet most of the mentioned criteria and the adoption of hAECs in cell therapy and regenerative medicine do not raise any ethical concern. Even if at present there are no clinically approved treatments of diabetes based on the use of stem cells, several therapeutic approaches have already been evaluated or are being evaluated in clinical trials [33].

Omnitrope® is a recombinant human GH approved for the treatment of children with growth disorders associated with short stature (e.g. GH deficiency, Turner syndrome, chronic renal insufficiency). PAtients TReated with Omnitrope® study assessed the safety and effectiveness of Omnitrope® among 186 children diagnosed with conditions of short stature requiring GH treatment [34]. Omnitrope® was associated with improvement in height standard deviation scores and height velocity standard deviation scores. Adverse reactions were reported in 9.1% of patients and two of these were considered serious (minimal increase in a known residual craniopharyngioma, and gait disturbance). However, conflicting data have been reported on the risk of developing cancer in children treated with GH [35]. Comparable results on Omnitrope® safety and efficacy have been achieved in a multicenter, retrospective follow-up study. A Spanish population with GH deficiency showed adult height within the local normal ranges and no adverse events during the GH therapy [36]. Consistently, the use of Genotropin®, a biosimilar product, has been studied in an open-label, multicenter, randomized trial. After 4 years of treatment, 316 children with idiopathic short stature in all treatment regimens achieved similar average height gains; however, the individualized dosing regimen utilizing less GH reached an equivalent height gain [37].

### Gastroenterology. 1-Celiac disease screening and manifestations; 2- functional gastrointestinal disorders

Celiac disease (CD) is an immune-mediated enteropathy caused by permanent intolerance to gliadin and related proteins present in gluten among genetically susceptible individuals. There has been an apparent increase in the incidence of CD over the past 30 years [38]. It is unclear whether the changing prevalence of reported cases of CD is due to a real increase in the number of cases, enhanced awareness of disease or more reliable serologic testing. The definitive diagnosis of CD is based on typical changes in the small intestine biopsy specimens. To screen individuals for CD serum tTG-IgA and EMA IgA antibodies are used. AGA determination has been necessary for children younger than 18 months of age who frequently present negative EMA and anti-tTG antibody results. Di Tola et al. [39] assessed the role of fecal celiac intestinal antibody tests in identifying CD patients. To this aim they compared a spot stool sample obtained from twenty-five sera EMA/anti-tTG positive CD patients and 12 sera EMA/anti-tTG negative controls, while they were eating gluten. They found that fecal IgA anti-tTG, IgA anti-DGP, IgA/IgG anti-tTG/DGP, and IgA anti-actin antibodies levels in CD patients were significantly higher in comparison with those detected in healthy controls. Fecal IgA EMA were found in 11 out of 25 (44.0%) CD patient but in none of healthy volunteers (*p* = 0.0066). Conversely, EMA IgG, anti-tTG IgG, and anti-DGP IgG were not detectable in fecal supernatants neither from CD patients nor from controls. These findings extended the celiac intestinal antibody pattern to other antibodies detectable in fecal supernatants but, for the small size of the sample, larger studies are needed to assess the role of fecal antibody tests in identifying CD patients, when serologic tests are negative. CD presents a wide range of both gastrointestinal and non-gastrointestinal symptoms. Khatib et al. [40] retrospectively evaluated clinical features in newly diagnosed children with CD. Among 165 subjects, the median age at presentation was 10.7 ± 4.3 years. No patient was less than 1 years of age. Abdominal pain was the most common presenting feature (52.7% of patients), followed by constipation (38.9%), diarrhea (31.1%), family history of first-degree relative (28.1%), diabetes mellitus type 1 (22.2%), failure to thrive (21.8%), reflux (15.1%), vomiting (14.5%), fatigue (9%), short stature (5.4%), thyroid disease (5.4%), Down syndrome (4.8%). All the patients had positive tTG-IgA, except 2. They found no correlation between the serum tTG-IgA and Marsh score, this suggests that tTG-IgA levels may not reflect the disease severity. This study shows a change in CD clinical presentation: the so-called classical symptoms of diarrhea, irritability and weight loss were less frequent and children more commonly presented with abdominal pain and constipation. This probably reflects rapid westernization leading to changes in dietary habits and early exposure to gliadin through highly processed foods. In conclusion, the possibility of CD should be considered in any child presenting with chronic abdominal pain and constipation.

Higher frequency of CD has been described among patients with neurological diseases. Most case reports suggest an association between autistic spectrum disorders (ASDs) and CD or positive CD serologic test results, but larger studies are contradictory. In a retrospective study, Calderoni et al. [41] evaluated CD prevalence in 382 children with ASDs. Overall, CD or positive CD serology were detected in 10 of 382 children with ASD, the mean age was 41 months (SD: 15.2; range: 20–67). The authors compared these data to that found in the general population, using the data from the survey of Mustalahti et al. [42] which investigated the CD prevalence in four European countries, including Italy. They found a statistically significant difference (*p* value = 0.0131) in the prevalence of CD cases in their sample (2.62%; 95% CI:1.0–4.2) compared to the Italian pediatric population (1.1%; 95% CI: 0.7–1.5) with an age-range from 0 to 19 years. These findings indicate that an association between ASD and CD cannot be excluded.

Neurological symptoms including ataxia, neuropathy, vestibular dysfunction, seizures, migraine, and headache are reported in 10%–22% of patients with CD [43]. Nenna et al. [44] aimed to determine the prevalence of CD in children with recurrent headache, to detect any relationship between them, to characterize the clinical and epidemiological features and finally to investigate the effects of the gluten free diet on headache in patients with CD. They studied 883 subjects (481 females, median age 9.8 years, range 3–19). In cases with positive serology tests, duodenal biopsy was performed for confirmation of CD. Of 883 patients with headache, 18 (2.04%) were celiac. Among these, 7 children were already diagnosed as celiac and were on a gluten free diet at the time of neurologic evaluation, 11 children had positive serology tests and underwent to duodenal biopsy that confirmed the diagnosis of CD. Among the patients with a diagnosis of CD, the most frequent type of headache was migraine without aura (50%), followed by tension-type headache and migraine with aura (38.9% and 11.1% respectively). The duration of attack in 72.2% of children was 30 min – 4 h and most of patients had mild or moderate intensity of headache. The prevalence of CD in children with headache was significantly higher (2.04%, *p* = 0.034), than that reported in Italian general population (1.2%) [45]. Headache improved among all patients after gluten free diet. These findings indicate that diagnostic tests for CD might be useful in children with recurrent headache, especially among non-responders to common therapies.

Recent studies also suggest that migraine may be associated with other gastrointestinal disorders, including irritable bowel syndrome, inflammatory bowel syndrome and functional gastrointestinal disorders (FGIDs). Le Gal et al. [46] did a robust case-control study in the emergency departments of four European hospitals to establish the prevalence of FGIDs in children and adolescents with primary headaches and investigate a possible relationship with migraine. They compared the prevalence of ten FIGDs in three groups of patients aged 6–18 years (257 with migraine, 167 with tension-type headache, and a control group of 648 headache-free individuals). Headaches were diagnosed by a neurologist using the International Classification of Headache Disorders and FGIDs by investigators masked to headache diagnosis who used Rome III diagnostic criteria. They found a significant association between the presence of migraine and functional dyspepsia and abdominal migraine. Furthermore, there was an inverse association between migraine and functional constipation. No association was found between tension-type headache and FGIDs. This study indicates a relationship between migraine and FGIDs. This is probably due to a shared pathophysiological mechanism of pain involving several inflammatory mediators, such as serotonin, and calcitonin gene-related peptide.

FGIDs are highly prevalent in childhood and pose a significant burden for the society, and affect the quality of life of patients and their families. FGIDs comprise a heterogeneous group of disorders with unclear pathophysiology. They are among the most challenging disorders to manage and many strategies, including pharmacological, dietary, cognitive behaviors and alternative approaches have been evaluated for their treatment. Salvatore et al. [47] systematically reviewed evidences on the effects and clinical relevance of pharmacological therapy in infants with FGIDs. They did not find improvement of regurgitation by proton pump inhibitors, anti-H2 or domperidone and they suggested considering alginate in infant regurgitation when symptoms are severe, associated with marked distress, and other non-pharmacological measures failed. For cyclic vomiting syndrome, a migraine variant characterized by recurrent, stereotypical episodes of intense nausea and vomiting, separated by intervals of wellness, they suggest intravenous infusion of fluids, ondasetron, lorazepam and acid inhibitors during the acute phase of vomiting. Several retrospective studies showed efficacy of cyproheptadine, propanolol and pizotifen in prophylaxis. In case of constipation [48], lactulose is recommended for infants younger than 6 months of age, whereas in older infants and in children polyethylene glycol with or without electrolytes represents the first-line therapy for both fecal disimpaction and maintenance. No evidence of effectiveness of pharmacological treatment were found for infant rumination syndrome, infant colic, functional diarrhea and infant dyschezia.

However, Madani et al. [49] recently demonstrated a good safety and efficacy profile in the use of cyproheptadine, a first-generation antihistamine with a serotonin antagonist effect, in children with FGIDs defined by Rome III clinical criteria. Complete improvement of symptoms was reported in 110 out of 151 patients (73%) (complete improvement group: CIG), 41 (27%) has partial or no improvement (improvement group/partial improvement group: NIG/PIG), it was completely effective in 36/55 (65%) children with functional abdominal pain and in 26/34 (76%) patients with functional dyspepsia. The mean duration of treatment with cyproheptadine was 8.91 months (range 1–48) in the CIG and 5.72 (range 1–26) in the NIG/PIG. Side effects were experienced by 49/151 patients. A higher proportion was in the NIG/PIG (41%, 20/49), presumptively for the higher doses needed. Sleepiness and weight gain were the most frequent adverse effects (19/151 (12%) and 15/151 (10%) respectively. Logistic regression analysis showed that lower the body weight, the higher were the odds of no or partial improvement. This is probably due to lower maturity of enterochromaffin cell receptors in the gut epithelium, suggesting caution in the use of cyproheptadine in children with low body weight. The single best predictor of clinical improvement was body mass index.

### Infectious diseases. 1-Brucellosis; 2-fever treatment; 3-pain in vaccination

Management, treatment and prevention of common and uncommon infectious diseases have been explored last year. Brucellosis is a serious public health problem that brings about socioeconomic consequences in many countries. The infection occurs in humans by ingestion of animal milk or its products, by direct contact with infected animals or inhalation of infectious aerosols, resulting in acute or chronic disease with non-specific symptoms, difficult to diagnose and treat. Up to 80% of patients develop rheumatic problems with subsequent functional impairment. Aktar et al. [50] conducted a retrospective study in 64 children to evaluate the role of inflammatory markers in the diagnosis of pediatric Brucella arthritis (BA). The diagnosis of arthritis was made on clinical symptoms (joint pain, restriction of movement, and swelling), isolation of Brucella from blood or synovial fluids and positive Brucella serology test >1:160. White blood cell (WBC) count, neutrophil count, lymphocyte count, hemoglobin (Hb), platelet count (PLT), mean platelet volume (MPV), neutrophil to lymphocyte ratio (NLR) and platelet to lymphocyte ratio (PLR), C-reactive protein (CRP), ESR, agglutination assay and culture for both blood and joint puncture. They found a significantly difference in WBC, Hb, neutrophil count, lymphocyte count, PLT, MPV, NLR and PLR values between the study and the control groups, but no correlation was found between MPV, ESR and CRP values. NLR, PLR and MPV mean values were higher in patients with BA compared to the control group (NLR 4.1 vs 1.4, *p* < 0.001; PLR 154 vs 106, *p* < 0.001; MPV 8.2 vs 7.3, *p *= 0.026). The most frequently involved joint was ankle and the most used antibiotics were rifampicin plus sulfamethoxazole/trimethoprim and gentamicin. Authors suggested the use of NLR and PLR ratios as reliable inflammatory markers in the diagnosis and follow-up of BA in children.

Karaman et al. [51] retrospectively analyzed the hematologic findings of 622 Turkish children, mean age 11.06 years (±4.3) with acute brucellosis. All cases had direct contact with sick animals, sick family member or consumption of unpasteurized dairy products from farms where brucellosis had been already established. Laboratory abnormalities were observed in 292 patients (46.9%). The most frequent laboratory findings in affected children included anemia (Hb <11 g/dL) in 178 (28.6%), thrombocytopenia (<150,000/mm3) in 100 (16%) and leukopenia (<4000/mm3) in 87 (13.9%) patients. Pancytopenia (Hb < 11 g/dL, PLT < 150,000/mm3, WBC < 4000/mm3) was observed in 48 (7.7%) children. The authors underlined the importance of establishing a prompt diagnosis of brucellosis in children with clinical manifestations, anemia, leukopenia, thrombocytopenia, or pancytopenia and positive epidemiological data, especially in rural parts of the country.

Current approaches for distinguishing bacterial from viral causes of acute infections are unreliable and inaccurate. No single biomarker distinguishes between viral and bacterial etiologies in settings where all may be involved. Van Houten et al. [52] aimed to assess the accuracy of a novel host–response-based diagnostic assay, ImmunoXpert, to distinguish bacterial infections from viral infections compared with CRP and procalcitonin. ImmunoXpert integrates the concentrations of three biomarkers: tumour necrosis factor-related apoptosis-inducing ligand (TRAIL), interferon gamma induced protein-10 (IP-10), and CRP. They conducted a prospective, double-blind study and recruited children, aged between 2 and 60 months, with fever without source or lower respiratory tract infection, consecutively presented at the emergency departments and pediatric wards. They found that the combination of CRP, IP-10, and TRAIL improved diagnostic ability to distinguish bacterial from viral infections and may help in rational use of antibiotics in children.

In the last decade thanks to progress in molecular technologies, newly discovered viruses have been identified. In this context, establishing a causal link between a newly identified virus and the disease as well as an association between mixed infections and an increase in disease severity can be challenging. Cebey-López et al. [53] conducted a prospective observational multicenter study to analyze the relationship between viral or bacterial co-infection detected by molecular methods, and the clinical phenotype of children with lower tract acute respiratory infections (LT-ARI). They enrolled two independent patient groups, 204 children in Spain (main group) and 97 children in the United Kingdom (replication group), under 14 years of age with a lower respiratory tract illness of sufficient severity to warrant admission to hospital, and a virus identified on nasopharyngeal swab or aspirate sample. Children presenting a bacterial superinfection had more severe respiratory distress, a higher severity score, required significantly more respiratory support and they had a longer hospital stay. Conversely, the number of detected viruses did not correlate with any markers of severity and did not influence the outcome. In addition, patients who received the pneumococcal vaccine had lower risk of being admitted to pediatric intensive care unit and had less risk of respiratory support requirement.

Regarding management of infectious diseases, causal treatment and antipyretics are the most commonly used drugs. The Italian Pediatric Society recently updated the guidelines for management of fever in children [54]. It highlighted that antipyretics should be administered orally with the purpose to control the child’s discomfort. Combined use of acetaminophen and ibuprofen is discouraged. Antipyretics are not recommended to prevent fever and local reactions in children undergoing vaccination, or febrile convulsions. Incorrect use of antipyretics is associated with fever phobia phenomenon that considers fever as health dangerous. According to a Portuguese study, fear of fever has been shown to be caused both by parents’ and healthcare professionals’ attitudes and beliefs [55]. A cross-sectional study analyzed the appropriateness of antipyretic dosages in a population of 1397 children with fever requiring a primary care outpatient visit [56]. Acetaminophen was administered for body temperature ≤ 38.4 °C in 74% of cases, with a daily overdose (>90 mg/kg/day) in 24% of cases. Self-medication was described in 60% of children, in all cases acetaminophen was administered at temperature < 38 °C. Self-prescription phenomena strictly related to the administration of high dosages of acetaminophen and was more frequent in caregiver with a higher level of education. Moreover, the intake of the drug as drops or syrup was associated with over dosage. Lubrano et al. [56] also emphasized the preventive role of the general practitioners in the education for the right prescription and assumption of acetaminophen in childhood.

Acetaminophen administration at standard as-needed doses is considered safe. However, it has been suggested that acetaminophen consumption may be associated with increasing asthma exacerbations, leading some physicians to avoid it in asthmatic children [57]. A multicenter, prospective, randomized, double-blind trial showed no difference in the number of asthma exacerbations, days with controlled asthma, use of salbutamol rescue inhaler or unscheduled visits to healthcare facilities for asthma, between children with mild persistent asthma treated with paracetamol and those with ibuprofen [58]. This trial did not address whether the use of acetaminophen or ibuprofen, as compared with no drug use, can worsen asthma [59].

Prevention of infective diseases is based on vaccinations. Unfortunately, vaccinations are a painful procedure. This pain may hamper their diffusion and may cause short term side effects, including immunological, endocrine, physiological and biochemical changes, and long-term side effects, such as distress and negative effects on the development of the central nervous system. Nowadays, various methods are used to reduce intensity and duration of painful stimuli and make medical procedures less painful. These include the injection technique, such as not aspirating; physical interventions, like positioning the child upright or breastfeeding; pharmacologic interventions, such as topical anesthetics and psychological interventions, like distraction techniques [60]. Flick application was used to determine if pain level and duration of crying were reduced in a cohort of infants receiving hepatitis B vaccine injection. A significant difference of pain score average was detected during and after the application between intervention group and controls. No difference was found on crying duration [61].

Breastfeeding may help reducing behavioral pain responses during vaccination compared to no treatment, administration of water, and other interventions such as cuddling, oral glucose, topical an aesthetic, massage, and vapocoolants [62].

Henoch-Schönlein purpura (HSP) is a systemic leukocytoplastic vasculitis characterized by IgA immune deposits and represents the most common vasculitis in childhood. Its etiology and pathogenesis remain unknown, nevertheless several genetic, immunological and environmental factors have been suggested as triggers. Da Dalt et al. investigated retrospectively in a case-control pediatric study the relation between drug or vaccine administration and HSP occurrence in 11 Italian centers [63]. Measles-mump-rubella vaccine was associated with an increased risk for HSP. However, vaccinated cases were only 8, suggesting a very low absolute risk of the condition in children who received measles-mump-rubella vaccine. No drug administration was associated with HSP.

### Neonatology. 1- Glucose monitoring; 2- salbutamol for respiratory distress syndrome; 3 oxygen saturation; 4-antibiotic consumption

Several advances relating to hypo-glycemia, oxygen treatment and antibiotic use in newborns have been made last year. Maintenance of euglycemia is especially challenging in the sick or low birth weight newborns. Prompt treatment can prevent permanent damage and promote intact survival. A continuous glucose monitoring system (CGMS) can improve glucose control and decrease the frequency of blood samples taken from newborns. In their study, Tiberi et al. [64] pointed to clarify accuracy and safety of a new CGMS in preterm infants using a Clarke error grid specifically modified for this population. They collected data from 20 preterm infants at increased risk for neonatal dysglycemia. The CGMS sensor detects the interstitial glucose concentration every 10 s and transmits wirelessly it to the monitor that calculates the average of glycemia every 5 min. Values <40 mg/dl or >430 mg/dl cause an alarm sound from the monitor. Authors’ findings confirmed a good correlation between CGMS and finger-stick capillary blood. Furthermore, the application of the sensor did not lead to any interference with nursing care and infants were not disturbed. Although the small population, this study proved the efficacy of the CGMS that can be used to quickly treat dysglycemia in preterm infants and avoid undetected pathological glucose fluctuations.

Hegarty et al. [65] conducted a randomized, double-blind, placebo-controlled study to determine whether 40% dextrose gel prevents neonatal hypoglycemia and hence reduces admission to Neonatal Intensive Care Units (NICU) in 416 babies at risk for hypoglycemia (preterm, diabetic mother, small or large for gestational age). Authors found that babies assigned to receive dextrose gel at any dose were less likely to develop hypoglycemia than those who received placebo. Gel was well tolerated and no babies developed hyperglycemia. Admission to NICU for hypoglycemia tended to be less common in babies randomized to dextrose gel, but no difference was found between dextrose and placebo groups. This study shows that a single dose of 200 mg/kg (0.5 ml/kg) of dextrose is effective and well-tolerated to reduce the incidence of neonatal hypoglycemia in babies born at risk. Harris et al. [66] reported data of a two-years follow-up study in 184 children who had been hypoglycemic (< 2.6 mM [45 mg/dL]) in the first 48 h of life and randomized to either dextrose (90/118, 76%) or placebo gel (94/119, 79%) to determine their neurodevelopmental outcome. All parents or caregivers of babies enrolled in the previous study (the Sugar Babies Study) [67] were invited to participate in this follow-up study, of the 237 eligible babies assessed previously, 184 (78%) were assessed at two years. Rates of impairment were similar in both treatment groups (dextrose 34/90 [38%] vs. placebo 32/94 [34%], *p* = 0.60). No differences were observed in rate of neurosensory impairment, processing difficulty, developmental delayed and vision problems. Authors demonstrated that the use of dextrose gel in treatment of hypoglycemia in neonatal period is not associated with adverse effects at two years’ corrected age.

Neonatal respiratory distress syndrome (RDS) due to surfactant deficiency is associated with high morbidity and mortality in preterm infants. In addition, in newborns with RDS there is an increase of lung fluid content and secretion of fluid derived from the blood, this contributes to respiratory distress. Lung airways fluid absorption is mediated via Na+/K + −ATPase channel. β-agonists increase the Na channel activity mediated by cAMP-PKA and lung sodium transport. Dehdashtian et al. [68] investigated the efficacy of intratracheal salbutamol administration in addition to surfactant in newborns with RDS. They assessed as primary outcome a decrease of Intubation-Surfactant administration- Rapid Extubation (INSURE) failure rate defined as the need for reintubation and mechanical ventilation within 72 h of extubation. They blindly randomized 48 newborns with RDS to receive salbutamol (interventional group) 0.2 mg/kg or saline (control group) 0.5 ml/kg. Surfactant was administered to the infants within 2 h of birth at initial dose of 2.5 ml/kg and subsequent doses of 1.5 ml/kg, according to the INSURE protocol. An INSURE failure was found in 10 of 24 neonates in salbutamol group compared to the 16 of 24 neonates in control group (*p* = 0.082). Authors found that salbutamol accompanied by surfactant reduced duration of hospitalization in newborns with RDS, but it did not change duration of oxygen therapy, duration of NCPAP, need of mechanical ventilation, mortality or other complications.

High-flow therapy is a recent popular approach of respiratory support in newborns. However, the evidence of its efficacy and safety has not yet been established. Roberts et al. [69] conducted an international, multicenter, randomized, noninferiority trial, to assess the efficacy of high-flow therapy as the primary respiratory support for preterm infants with respiratory distress. They recruited 515 infants born at a gestational age of 28 weeks 0 days to 36 weeks 6 days from nine NICU. After reviewing the primary outcome data (treatment failure within 72 h after randomization), the steering committee stopped recruitment since high-flow treatment resulted in a significantly higher rate of treatment failure than CPAP when used as primary respiratory support for preterm infants at any gestational age (71/278 infants (25.5%) in the high-flow group and 38/286 infants (13.3%) in the CPAP group; *p* < 0.001). Authors highlighted that high-flow therapy as first line respiratory support in preterm infants is not recommended.

Although oxygen is crucial for survival, too much oxygen can be harmful. Monitoring and maintaining optimal oxygen saturation (SpO2) by pulse oximetry is a critical component of treating respiratory diseases. Mascoll-Robertson et al. [70] determined whether the histogram distribution of SpO2 over a 24-h period predicts readiness for weaning respiratory support in preterm infants. Their hypothesis was that infants with > 15% of time spent with SpO2 < 86% before transitioning from CPAP or high-flow nasal cannula (HFNC) to low-flow nasal cannula, oxyhood, or room air are more likely to fail transitioning. Thirty-five preterm infants with gestational ages of 24–32 weeks were enrolled in this prospective, observational cohort study. The histogram distribution of pulse oximetry showing the percentage of time spent in the preset oxygen saturation ranges (96–100, 91–95, 86–90, 81–85, and < 80%) for a 24-h period was recorded daily and the clinical team was blinded to the 24-h histogram distributions. Overall, 7 of 31 subjects failed transition from CPAP/HFNC. In the failure group, 2/7 (29%) subjects experienced SpO2 < 86% ≥ 15% of the time before transitioning compared with none in the successful group (*p* = 0.045). The failure group experienced SpO2 < 86% 11 ± 12% of the time pre-wean compared with 3 ± 5% of time in the successful group (*p* = 0.02). Conversely, infants in success group experienced a greater percentage of time with SpO2 > 95% compared with the failure group. These findings suggest that the combination of pulse oximetry histogram and clinical stability criteria can help clinicians to determine readiness for invasive respiratory weaning, preventing transition failures.

Newborns are a population in which antibiotic consumption is extremely high. Targeted antibiotic therapy should help to reduce antibiotics consumption. Cantey et al. [71] conducted a two-phases study, a retrospective phase followed by an interventional phase to safely reduce antibiotic use in the NICU. In the first months of the study, all antibiotic use in infants admitted to the NICU was recorded. During the intervention period, empirical antibiotic therapy was set to discontinue after 48 h and the duration of therapy for pneumonia and culture-negative sepsis was limited to 5 days. A significant decrease in antibiotic consumption, defined as days of therapy per 1000 patient-days, was found in the intervention period in comparison with the baseline period (343.2 days of therapy per 1000 patient-days at baseline vs 252.2 days of therapy per 1000 patient-days during the intervention; *p* < 0.0001). No difference in the proportion of infants who were colonized with multidrug-resistant organisms between baseline and intervention period was documented. There was no difference in the outcome of late-onset sepsis, necrotizing enterocolitis, or death in infants 32 weeks’ gestation or younger in the baseline period (17.1%) compared with the intervention period (15.8%). The intervention resulted in both a 27% decrease in total antibiotic use and a higher proportion of antibiotic use directed at proven infection or necrotizing enterocolitis. These findings suggest that an overall increase in appropriate antibiotic administration in NICU can be designed with close monitoring of patient safety outcomes.

### Nephrology. Rituximab and antithrombotic prophylaxis in nephrotic syndrome

Articles provided novel information on nephrotic syndrome, a common and important disorder characterized by heavy proteinuria and hypoalbuminemia, usually associated with dyslipidemia and hypercoagulability [72]. International guidelines recommend a low-dose alternate-day steroid regimen as first-line treatment for the management of children with frequently-relapsing or steroid-dependent nephrotic syndrome [73, 74]. Children with nephrotic syndrome may have clinical relapses and/or develop steroid dependence, this being defined as complicated frequently relapsing/steroid-dependent nephrotic syndrome. Resistance to steroids and all immunosuppressive agents may also occur in a proportion of affected patients, and in these cases refractory steroid-resistant nephrotic syndrome, a condition with substantial risk of end-stage renal failure, has been described. Maratea et al. [75] carried out an analysis aimed at assessing the efficacy and safety of rituximab, a chimeric anti-CD20 monoclonal antibody, for the treatment of children with nephrotic syndrome. The inclusion criteria of the studies were: a) a study population of children with complicated frequently relapsing/steroid-dependent nephrotic syndrome; b) the choice of rituximab as treatment; c) the choice of steroids and/or calcineurin inhibitors as control therapy; d) complete remission rate as end-point. Four studies were included in the meta-analysis, having as end-point the percentage of patients in remission at 6 months. The intervention group consisted of patients treated with rituximab *plus* prednisone and/or calcineurin inhibitors, while controls were treated only with prednisone and/or calcineurin inhibitors. The end-point was the percentage of patients in remission at 6 months. Other end-points (e.g., relapse-free survival rate for efficacy and adverse events for safety) were also reported. Pooled data from these studies favored the use of rituximab (RR 5.25; *p* < 0.0001). These results were confirmed also at 12 months in two of the four studies. Moreover, children treated with rituximab had a limited number of adverse effects. Although more patients in the rituximab group had serious adverse events (mainly hypoproteinemia, lymphocytopenia and neutropenia) compared to controls, the difference was not significant (*p* = 0.36). The strength of this meta-analysis is that authors include a total of 101 children from the four studies, whereas other studies include an extremely limited study population. The results of this meta-analysis confirm a significant incremental benefit of adding rituximab to corticosteroid and/or calcineurin inhibitors for the treatment of nephrotic syndrome.

Artoni et al. [76] described an adolescent with a steroid-dependent nephrotic syndrome who experienced a central vein thrombosis during the relapse of his baseline disorder. Authors detected a severe inherited thrombophilia (inherited dysfunctional protein S deficiency) that was considered as an important additional risk factors for thrombosis. Eventually, the patient underwent a successful course of systemic thrombolysis plus anticoagulant therapy. Venous thromboembolic events are increasing in children because of therapeutic advances in primary illnesses that previously caused mortality (congenital heart disease, malignancy, trauma) [77]. However, the estimated incidence of symptomatic vein thrombosis in children is significantly less than that in adults [78]. The etiology includes thrombophilia, head and neck infections, and systemic illness. Among the acquired prothrombotic disorders, nephrotic syndrome may be complicated by thromboembolic events [79]. Thrombotic events during infancy and childhood are increasingly recognized as a significant source of mortality and morbidity. Thrombophilia can be defined as a predisposition to form clots inappropriately. A hereditary thrombophilia results when an inherited factor, such as antithrombin or protein C deficiency, requires interaction with components that are inherited or acquired before onset of a clinical disorder. In the presence of ongoing risk factors, such as active nephrotic syndrome, therapy should continue until the risk factor has resolved, but its optimal intensity is controversial.

### Neurology. 1-Second generation antipsychotics; 2-Moebius syndrome. 3-Stroke; 4-migraine, 5-chromosomal imbalances in intellectual disability

The update by Pisano and co-workers concern about the safety of second generation antipsychotic (SGA) drugs in children and adolescents [80]. The manuscript was specifically focused on the studies published over a 5-years-period that report the effect of SGA on a considerable number of events or variables, i.e. body weight; abnormal blood glucose levels, and elevated lipid levels; prolactin concentration; and cardiometabolic and peripheric or central nervous systems parameters. SGA did not behave as a homogeneous group in children and adolescents with psychotic and mood disorders. Except for clozapine, the heterogeneity within the SGA group was mainly due to differences in the rates and severity of adverse events, especially regarding weight gain as a proxy for the risk of cardiometabolic disturbances [81]. Antipsychotic drugs block dopamine receptors and are used to manage psychosis as well as other mental illnesses that may or may not have psychotic features, such as bipolar disorders and major depressive disorder. First-generation antipsychotic drugs are more likely to cause adverse effects such as extrapyramidal symptoms and tardive dyskinesia. Adverse effects of SGA drugs typically were related to metabolic abnormalities such as weight gain, abnormal blood glucose levels, and elevated lipid levels. Neuroleptic malignant syndrome is a rare but serious adverse effect of antipsychotic drugs that causes mental status changes, hyperthermia, and generalized rigidity. Timely diagnosis is essential due to a high risk for related morbidities if the syndrome remains untreated. Some adverse effects of antipsychotics can be identified and managed so that patients can continue beneficial therapy while minimizing the physiologic consequences. Patients taking antipsychotic drugs should be monitored regularly for adverse effects.

Moebius syndrome (MBS) is a congenital, non-progressive facial and abducens nerve palsy in the presence of full vertical gaze and may be associated with limb abnormalities and craniofacial dysmorphisms. MBS is now defined as a disorder of encephalic development and recent gene discoveries have shown this to be a dominant disorder in a subset of patients. Accurate diagnosis and management by a multidisciplinary team with expertise in congenital facial palsy is paramount [82]. Picciolini and co-workers described a cohort of 50 MBS patients aged from 1 month to 14 years from Italy [83]. All subjects had previously undergone a multidisciplinary follow-up aimed at identifying the major complications which are associated to MBS. Findings from the ophthalmological, neurological, audiological, orthopaedic evaluation and from brain magnetic resonance imaging were presented. Nutritional impairment was found in 16% of the cases as some patients were fed by nasogastric tube until 1 year of age. A high proportion of cases were found to have alteration of VII cranial nerve, but normal intellectual quotient. Problems in language and hearing caused hearing loss in approximately 7% of patients, language delay in 31%, speech deficit in 42%.

MBS is more than a cranial nerve or nuclear disorder as lower brainstem involvement is described in affected patients. The diagnosis of MBS may be difficult in some patients with atypical signs of facial diplegia and other cranial nerve palsies. Once diagnosed, patients with MBS deserve a multidisciplinary follow-up [84].

Migraine and stroke are common events in the healthy population. A link between them has been proposed years ago, with various theories explaining it, but the reason for the association remains unclear. Unfortunately, migraine prophylaxis does not seem to decrease the risk of stroke [85]. Nevertheless, subjects with major cardiovascular risk factors should be encouraged to adopt migraine prophylactic strategies. Spalice et al. [86] briefly summarized the characteristics of the association, provided a classification of migraine-related stroke, reported on its pathogenesis and made recommendations on the diagnostic pathway also in childhood. Migraine is apparently a benign condition, while stroke remains an extremely common cause of disability, with a possible fatal outcome. Both disorders have a high socioeconomic burden. Migraine has been associated with both cardiac and vascular disorders, most of all hypertension and myocardial ischemic events, which may be complicated by stroke [87, 88]. Spalice et al. [86] also focused on the fact that genetic factors may play a role in the correlation between migraine and stroke. We need further investigation to better understand the complexity of migraine–stroke association and to make firm recommendations for the future.

Array-CGH (aCGH) is used in current routine clinical practice for diagnosis of patients with intellectual disability (ID), multiple congenital anomalies (MCA), and autism spectrum disorder (ASD) [89]. It can detect small chromosomal imbalances, copy number variations (CNVs), and can also closely define their size and gene content [90]. The aims of Cappuccio’s study were to establish clinical clues potentially associated with pathogenic CNVs and to identify cytogenetic indicators to predict the pathogenicity of the variants of uncertain significance in a cohort of 214 paediatric patients tested with aCGH either as first-tier test or as part of a wider diagnostic work-up. Pathologic findings were detected in 65 individuals (30%) and CNVs revealed the presence of a known syndrome in 46 cases. The CNVs were most frequently represented by deletions showing the highest gene-dosage content. The presence of ASD and a positive family history for ID, ASD and/or MCA were clinical pointers for the suspicion of a pathological chromosomal rearrangement together with eye anomalies, hearing loss, neurological signs, cutaneous dyschromia, and endocrinological disorders. Among patients carrying variants of uncertain significance, a higher gene density was found in patients affected by ID, suggesting the possibility of a pathogenic gene involved and causing ID in these patients. The detection of patients who are most likely to have a diagnosis by aCGH, minimizing the number of benign CNVs or negative results, remains an attractive goal [91]. This study confirms the usefulness of aCGH as first-tier test in patients with neurodevelopmental disorders. Furthermore, a diagnostic algorithm for patients with unexplained ID and/or MCA and/or ASD has been proposed.

### Nutrition. Safety of donor milk and infant formula

New insights have been provided into the safety of milk for infants. Holder pasteurization (HoP) is the most widespread method for the treatment of donor milk [92]. However, data from literature seem to suggest that HoP may cause degradation of some bioactive components [93, 94]. Peila et al. [95] carried out a study to determine the effect of HoP on the protein profile of human milk (HM) using the GeLC-MS method, a proteomic approach and a promising technique able to offer a qualitative HM protein profile. The protein profile before and after HoP showed qualitative differences only in 6 samples out of 20. The differences interested only colostrums and transitional milk samples and regarded the decrease of the electrophoretic bands corresponding to alpha and beta-casein, tenascin, lactoferrin and immunoglobulin. However, no significant differences were observed after correction for delivery mode and/or gestational age at birth. In the remaining 14 samples, no detectable differences were found even after correction for milk maturity degree. In most of, many of samples, HoP did not cause any modification, thereby preserving the biological activity of HM proteins. Future studies should be designed to investigate whether the observed differences are also confirmed by other techniques able to assess the protein changes due to thermic treatments including the interaction between proteins and sugars, or proteins and lipid, with possible toxic derivatives.

Powdered infant formulas (PIF) are generally not sterile and are often contaminated by several bacteria strains [96]. Among them, Cronobacter species, previously known as Enterobacter sakazakii, is one of the most harmful, since it might be the causative agent of sepsis and meningitis in newborns and preterm infants during the first weeks of life, with a mortality rate up to 80% [97]. Therefore, some precautions are required in the home handling and dilution of PIF. Although there is wide consensus about the need that a PIF should be used immediately after being diluted or, if not, stored at <5 °C, the optimal temperature of the water used to dilute PIF is still debated by scientific societies and health agencies. The current knowledge was reviewed in the paper by Silano et al. [98] that provides evidence to cautiously advise the use of hot water at a temperature of 70 °C in the dilution of PIF to prevent the Cronobacter species contamination and growth. Research is currently investigating methods to improve the microbiological safety of PIF, via the inclusion of protectants or alternative methods to lower or eliminate microbiological contamination. Among the most promising, bioactive peptides, organic acids, probiotics and prebiotics should be cited [99]. Supercritical carbon dioxide, gamma irradiation, exposure to microwaves or to a combination of UV and near infrared radiation heating are some among the possible alternative physical methods. However, all these recent technologies, though promising, still need further investigation on safety, reproducibility and cost/benefit ratio issues, before becoming standard procedures.

### Pulmonology. 1-Bronchial secretion clearance; 2-recurrent pneumonia

Management of secretions in children with cerebral palsy is often problematic due to impaired cough, severe deformation of the rib cage, and patients’ inability to collaborate with chest physiotherapy [100]. While several techniques allowing the management of airways secretions in a comfortable, noninvasive mode exist, they are nevertheless influenced by the patient’s cognitive status and ability to cooperate [101]. An efficient drainage of respiratory secretions through a “vacuum” effect has been found to be effective and safe in adult patients, but the effectiveness of such a treatment in children with cerebral palsy is still under debate [102]. Assessing the effectiveness of different methods and techniques of secretion clearance is hampered by the lack of direct outcome measures and by limited patient’s cooperation. Garuti et al. [103] evaluated the efficacy of Free Aspire in 8 patients with cerebral palsy who had experienced more than 3 episodes of respiratory exacerbations in the latest year despite therapeutic optimization (including bronchial clearance techniques) and who had received at least one antibiotic course or underwent at least one access to the emergency room or admission to hospital in the 6 months prior to the study. This Free Aspire device uses Vakűm technology to remove secretions from the bronchial tree in hyper secretive patients. During expiration, the airflow is accelerated by the Venturi effect inside a special connector, and the acceleration is proportional to the flow of air on spontaneous breathing, without requiring any cooperation. In the 6 months prior to start the treatment, patients had a mean number of 4.0 visits by the primary care pediatrician (PCP), spent 14 days in hospital, and received antibiotics for 35 days. After the first 6 months of treatment, they had 1.7 PCP visits, no days spent in hospital, and 9.75 days of antibiotic therapy. At 12 months of treatment, the number of PCP visits, days in hospital, and days of antibiotics were unchanged. At 18 months of treatment, no hospitalizations had occurred, PCP visits were 0.25, and days of antibiotic therapy were 4.8. The technique proved to be safe and well tolerated. The authors concluded that the application of Free Aspire to improve the bronchial secretion clearance in non-collaborating patients with cerebral palsy is feasible and effective in reducing respiratory complications and exacerbations, as well as the utilization of healthcare resources (PCP visits, days of hospitalization, need for antibiotics). Its regular use maintains its efficacy over time. Randomized controlled studies in larger patient cohorts on how to improve the mucociliary clearance and thus optimize the respiratory condition of children with cerebral palsy are required.

Recurrent pneumonia (RP) occurs in 6.7–8.2% of the general pediatric population in industrialized countries, is frequently described in association with a severe underlying disease favoring lung infections, and is considered a marker of the possible development of non-cystic fibrosis bronchiectasis [103]. However, RP are often found in children without significant risk factors, and this can delay the diagnosis of bronchiectasis, thus increasing the risk of worsening lung function during adolescence and adult life [104, 105]. The study by Patria et al. [106] was designed to analyze the clinical records of 42 children with RP (21 with non-cystic fibrosis bronchiectasis and 21 without non-cystic fibrosis bronchiectasis) to identify factors that may lead to an early suspicion of bronchiectasis, thus leading to prompt diagnosis and implementation of the best prophylactic and therapeutic measures. The most frequent underlying diseases in both groups were chronic rhinosinusitis with post-nasal drip and recurrent wheezing. FEV_1_ and FEF_25–75_ values were significantly lower in children with bronchiectasis than in those without. Bronchodilator responsiveness was observed in seven children with bronchiectasis and two without (*p* = 0.13). As reduced FEV_1_ and FEF_25–75_ values seemed to be associated with an increased risk of developing bronchiectasis in children with RP, the authors suggested the need for further studies to confirm the diagnostic usefulness of spirometry in such cases.

## Conclusions

Research in pediatrics was wide-ranging during this year. This review summarizes new evidences about physiopathology, diagnosis, prevention and treatment of common diseases in childhood. Several scientific societies have updated guidelines on various matters including fever, atopic dermatitis, nephrotic syndrome, gastrointestinal disorders, and hypertension. Overall, the management of pediatric diseases has been improving to date towards a patient’s tailored approach.

## Abbreviations

AAA: IgA anti-actin antibodies
aCGH: Array-CGH
AD: Atopic dermatitis
ASD: Autism spectrum disorders
CD: Celiac disease
CEG: Clarke error grid
CGMS: Continuous glucose monitoring system
CHDs: Congenital heart defects
CIA: Celiac intestinal antibody
CIG: Complete improvement group
CNVs: Copy number variations
CRP: C-reactive protein
cSLE: Childhood-onset systemic lupus erythematosus
CVS: Cyclic vomiting syndrome
EMA: Anti-Endomysium
ESPGHAN: European Society for Pediatric Gastroenterology, Hepatology, and Nutrition
ESR: Erythrocyte sedimentation rate
FAP: Functional abdominal pain
FD: Functional dyspepsia
FGIDs: Functional gastrointestinal disorders
GH: Growth hormone
GI: Gastrointestinal
Hb: Hemoglobin
HFNC: High-flow nasal cannula
HM: Human milk
HoP: Holder pasteurization
IBS: Irritable bowel syndrome
ID: Intellectual disability
INSURE: Intubation-Surfactant administration- Rapid Extubation
IP-10: Interferon gamma induced protein-10
IVIG: Intravenous immunoglobulin
KD: Kawasaki disease
LT-ARI: Lower tract acute respiratory infections
MBS: Moebius syndrome
MCA: Multiple congenital anomalies
MP: Mycoplasma Pneumoniae
MPV: Mean platelet volume
nCPAP: Nasal Continuous Positive Airway Pressure
NICU: Neonatal Intensive Care Units
NIG/PIG: No improvement group/partial improvement group
NLE: Neonatal lupus erythematosus
NLR: Neutrophil to lymphocyte ratio
PCP: Primary care pediatrician
PIF: Powdered infant formulas
PLR: Platelet to lymphocyte ratio
PLT: Platelet count
PPI: Proton pump inhibitors
RDS: Respiratory distress syndrome
RP: Recurrent pneumonia
SGA: Second generation antipsychotic
SLE: Systemic lupus erythematosus
SpO2: Oxygen saturation
TRAIL: Tumour necrosis factor-related apoptosis-inducing ligand
tTG: Transglutaminase
WBC: White blood cell
